# The Discovery of GALM Deficiency (Type IV Galactosemia) and Newborn Screening System for Galactosemia in Japan

**DOI:** 10.3390/ijns7040068

**Published:** 2021-10-25

**Authors:** Atsuo Kikuchi, Yoichi Wada, Toshihiro Ohura, Shigeo Kure

**Affiliations:** 1 Department of Pediatrics, Tohoku University School of Medicine, 1-1 Seiryo-machi, Aoba-ku, Sendai 980-8574, Japan; wada@med.tohoku.ac.jp (Y.W.); kure@med.tohoku.ac.jp (S.K.); 2 Division of Clinical Laboratory, Sendai City Hospital, 1-1-1, Asuto-Nagamachi, Taihaku-ku, Sendai 982-8502, Japan; tohura@med.tohoku.ac.jp; 3 Tohoku Medical Megabank Organization, Tohoku University, 2-1 Seiryo-machi, Aoba-ku, Sendai 980-8573, Japan

**Keywords:** neonatal screening, newborn screening, galactosemia, GALM, GALM deficiency

## Abstract

The Leloir pathway, which consists of highly conserved enzymes, metabolizes galactose. Deficits in three enzymes in this pathway, namely galactose-1-phosphate uridylyltransferase (GALT), galactokinase (GALK1), and UDP-galactose-4′-epimerase (GALE), are associated with genetic galactosemia. We recently identified patients with galactosemia and biallelic variants in *GALM*, encoding galactose epimerase (GALM), an enzyme that is directly upstream of GALK1. GALM deficiency was subsequently designated as type IV galactosemia. Currently, all the published patients with biallelic *GALM* variants were found through newborn screening in Japan. Here, we review GALM deficiency and describe how we discovered this relatively mild but not rare disease through the newborn screening system in Japan.

## 1. Introduction

### 1.1. Enzymes That Catabolize D-galactose

**Figure 1:**
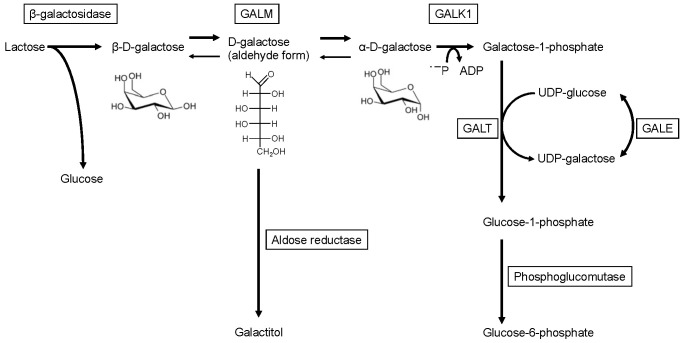
Enzymatic pathways related to galactose metabolism.

Lactose, the main energy source for human babies, is hydrolyzed into β-D-galactose and D-glucose on the intestinal microvilli by β-D-galactosidase and is absorbed into the blood. D-galactose is converted to glucose-1-phosphate via the Leloir pathway and further converted to glucose-6-phosphate, a precursor for the glycolytic, the pentose phosphate, and the gluconeogenic pathways (Figure 1) [1]. The Leloir pathway was named after its discovery by Dr. Louis Leloir in the 1940s [2,3,4]. It consists of highly conserved enzymes, including galactose-1-phosphate uridylyltransferase (GALT, EC 2.7.7.12), galactokinase (GALK1, EC 2.7.1.6), and UDP-galactose-4′-epimerase (GALE, EC 5.1.3.2). Since the 1950s, mutarotase, an enzyme that catalyzes the conversion between α and β-anomers of D-glucose, D-galactose, and some structurally related sugars, has been identified in various organisms [5,6,7]. Subsequently, human galactose mutarotase (or aldose 1-epimerase; GALM, EC 5.1.3.3) was isolated [8,9,10,11]. Although GALM catalyzes the equilibrium between β-D-galactose and α-D-galactose, GALM predominantly promotes the conversion from β-D-galactose, which is primarily derived from dietary lactose, to α-D-galactose in vivo. Since GALK1 (one step downstream of GALM) only utilizes α-D-galactose as a substrate, excluding β-D-galactose [12], GALM supplies α-D-galactose for GALK1. The four enzymes are encoded by a gene cluster in some organisms including *S. cerevisiae*, *Lactococcus lactis* [13], and *E. coli*, wherein these genes are cistrons of the gal operon [14]. This suggests a coordinated action of the enzymes.

### 1.2. Galactosemia Resulting from Enzyme Deficiencies in the Leloir Pathway

Mutations in three genes encoding enzymes in the Leloir pathway, namely *GALT* (MIM: 606999), *GALK1* (MIM: 604313), and *GALE* (MIM: 606953), have been associated with three genetic galactosemias: GALT deficiency (MIM: 230400), GALK1 deficiency (MIM: 230200), and GALE deficiency (MIM: 230350), respectively. GALT deficiency causes the most severe galactosemia (type I galactosemia, known as classic galactosemia), which is potentially lethal. If untreated, patients with severe GALT deficiency exhibit failure to thrive, sepsis (mainly due to *E. coli*), and liver failure during the neonatal period. Various chronic complications, including cataracts, intellectual/psychiatric disorders, movement disorders, and primary ovarian insufficiency, are also associated to classic galactosemia. Some of these chronic complications may occur despite life-long galactose restriction [15]. In contrast, cataracts are the only consistent symptom in patients with GALK1 deficiency (type II galactosemia). Recently, bleeding diathesis, encephalopathy, and an elevation of transaminase during the neonatal period have been reported to be associated with GALK1 deficiency [16]. GALE deficiency (type III galactosemia) has two subtypes: the “general” type [17] and the “peripheral” type [18]. The “general” type is extremely rare and resembles classic galactosemia. Most GALE deficiencies consist of the “peripheral” type, in which the deficiency is restricted to red and white cells, and has virtually no symptoms. GALE deficiency, however, is considered a continuum disorder rather than a binary condition [19] and it has also been associated with thrombocytopenia [20,21].

### 1.3. GALM Deficiency and Galactosemia in Humans

In an aqueous solution, galactose spontaneously equilibrates between α-D-galactose and β-D-galactose [22], but the conversion rate is slower when it is bound to protein [14,23]. Deletion of human GALM homologs is not fatal but results in slow growth in some organisms, including yeast [23] and *E. coli* [14]. Although human GALM deficiency has not been described, we recently identified eight patients with galactosemia and biallelic variants in the *GALM* gene [10,11] (MIM 137030) [24]. The entity was later designated as type IV galactosemia (MIM 618881) [25]. Here, we review GALM deficiency and discuss how we discovered this relatively mild disease through the newborn screening system in Japan.

## 2. Clinical Features of GALM Deficiency

At the time of newborn screening (NBS), the levels of galactose were mildly elevated (8.7–15.7 mg/dL; cut-off value, 3–6 mg/dL) in GALM-deficient patients. The maximum galactose levels were 17.3–41.9 mg/dL during follow-up. This elevation of galactose appears to be mild compared to that observed in GALK1 deficiency, in which galactose (and total galactose (galactose + galactose-1-phosphate)) levels in the blood reach up to several hundred mg/dL in some patients [16]. It is worthy to note that galactose-1-phosphate (Gal-1-P) was detected during the neonatal period (0.3–10.8 mg/dL at NBS; cut-off value, 10–15 mg/dL), although the levels did not exceed the cut-off value in most cases. Gal-1-P levels declined after the neonatal period. Interestingly, this transient fluctuation of Gal-1-P is also observed in GALK1 deficiency [16] and these patterns of galactose and Gal-1-P during the neonatal period are similar for the two types of galactosemia. The same mechanism may be responsible for the Gal-1-P fluctuation during the neonatal period in both GALM and GALK1 deficiencies. However, further studies are needed, e.g., concerning the consideration of technical issues during the measurement of Gal-1-P.

Cataracts are the only reported persistent symptom and were observed in two patients. In one patient, cataracts resolved during a galactose restriction diet. In galactosemia, cataracts develop from a build-up of galactitol in the eye lens. Galactitol is produced from an excess of galactose, which is catalyzed by aldose reductase. The substrate of aldose reductase is neither of the two anomers of D-galactose (pyranose form) but is the aldehyde form (chain type) of D-galactose, which is their intermediate (Figure 1) [25,26]. While alpha-D-galactose accumulates during GALK1 deficiency and beta-D-galactose is likely to accumulate in GALM deficiency, both anomers are likely to produce galactitol through the intermediate (aldehyde form).

Other complications, such as those observed in classic galactosemia, have not been reported in GALM-deficient patients. For example, neurological complications, including intellectual disability, speech disorder, and ataxia, were not observed in patients with GALM deficiency. Additionally, no patients with primary ovarian insufficiency were reported, although more data on GALM-deficient adults are needed.

Galactose intake was restricted in all the reported patients. Among them, two patients were able to stop galactose restriction at the last follow-up. This may have been possible because the amount of lactose intake according to body weight decreases with age. Spontaneous equilibration without GALM may be sufficient for the metabolism of a small amount of β-D-galactose, at least in some toddlers or older patients with GALM deficiency.

## 3. Molecular Genetics of GALM Deficiency

Currently, five pathogenic *GALM* variants have been identified. Three variants consist of nonsense and frameshift variants (p.Arg82*, p.Ile99Leufs*46, and p.Trp311*), leading to premature termination codons. The other two variants represent missense variants (p.Gly142Arg and p.Arg267Gly). In the eight Japanese patients reported, p.Gly142Arg and p.Ile99Leufs*46 were prevalent and accounted for 44% (7/16) and 25% (4/16) of the variants, respectively. The two residues, Gly142 and Arg267, affected by the missense variants are distant from the sugar-binding site [11,24,27]. Based on the results of in vitro GALM expression and protein stability assays, all five variant proteins, including the two mutant proteins with missense mutations, were unstable compared to the wild-type proteins.

## 4. The Estimated Prevalence of GALM Deficiency and Reports from Other Countries outside Japan

To date, all reported patients harboring biallelic *GALM* variants were identified through the NBS in Japan. The existence of individuals with biallelic pathogenic variants in *GALM* in other populations is corroborated because some pathogenic variants, including the variants found in patients with GALM deficiency (such as p.Arg82* and p.Gly142Arg), are present in public genome databases representing various ethnicities. Among 67 variants that were prevalent in the ExAC database, we considered 30 variants to be pathogenic based on in vitro expression and enzymatic assays to estimate the incidence of GALM deficiency [28]. Based on the prevalence of these pathogenic variants, the incidence was estimated to be 1:228,411 in all populations, 1:80,747 in the Japanese population, and 1:10,388 in the African population (the highest population). The estimated incidence of GALM deficiency is likely comparable to that of the GALT, GALK1, and GALE deficiencies [29,30,31,32,33]. Consistently, we identified several patients with GALM deficiency in Japan after the first report (unpublished). No additional reports, however, have been reported from countries outside Japan, although we have been notified of a few patients with biallelic pathogenic variants in *GALM* through personal communications.

## 5. The Reasons why the First Report of GALM Deficiency Originated from Japan: Different NBS Systems and a Wide Range of Phenotypes

It is interesting to speculate as to why the first report of GALM deficiency originated from Japan. One possible factor is the differences in NBS systems between countries. In many Japanese NBS systems, galactose, Gal-1-P, and total galactose levels, as well as GALT activity, are measured during first-tier screening. Thus, the patients with GALM deficiency are identified if they exceed the threshold of galactose levels, while GALT activity is normal and the levels of Gal-1-P are usually below the threshold. They are then subject to closer examination. Published patient data indicate that total galactose levels are higher than the NBS cut-off values for classic galactosemia (7–10 mg/dL). Therefore, it may also be possible to identify GALM deficiency by total galactose levels. Among these cases, the patients with GALM deficiency would be identified after other causes are ruled out, including GALT/GALK1/GALE deficiencies, portosystemic shunt, and citrin deficiency.

Some countries do not screen NBS for galactosemia. In Europe, screening for classic galactosemia is done in approximately one-third of the countries (20/51, 39.2%) but not in the others (Table 1) [34].

Even in countries in which galactosemia is a screening target, the primary goal of NBS is to screen for classic galactosemia, which is the most severe type of galactosemia, and not GALM deficiency. NBS systems for classic galactosemia often contain only GALT activity and Gal-1-P, thus patients with GALM deficiency will be missed. In the US, NBS for GALT deficiency has been deployed in all states and territories. The enzymatic activities of Gal-1-P and GALT are used during first-tier screening in most states. Galactose is not used in all states and only half of the states (25/53, 47.2%) use total galactose levels (https://www.newsteps.org/; accessed on 25 June 2021 Table 1). In particular, as a first-tier screening test, total galactose level testing is performed in only a quarter of the states (12/53, 22.6%). Overall, most NBS systems for classic galactosemia in the US would not identify GALM deficiency.

The situation in which NBS is used to detect other diseases for classic galactosemia is the same as that for GALK1 deficiency. In several countries, NBS for GALK1 deficiency has been performed using total galactose levels [32,35]. It may be possible to incorporate screening for GALM deficiency by measuring galactose or total galactose levels in NBS for classic galactosemia. In addition, since several states and territories in the US have adopted the *GALT* mutation analysis as a second-tier test, it may be useful to expand the scope to other galactosemia-associated genes, including *GALM*, as another possible extension of neonatal screening for galactosemia.

In countries where GALM deficiency cannot be detected by NBS, the mild phenotype of the disease may make it even more difficult to diagnose. Currently, no permanent sequelae such as systemic complications or intellectual disability, which are observed in type I galactosemia, have been reported, except for cataracts. Therefore, in NBS-negative babies without cataracts, the presence of GALM deficiency would not be noticed even if they had elevated blood galactose levels. It has also been suggested that the phenotype becomes milder as the amount of lactose intake according to body weight decreases in older children. In a study of eight GALM-deficient patients, two were able to discontinue their dietary restrictions. Some of these cases may have also been diagnosed as “transient” unexplained galactosemia or mild GALK1 deficiency based on mildly elevated Gal-1-P during the neonatal period [16]. Thus, it is likely that a significant number of patients with biallelic pathogenic variants with *GALM* remain undiagnosed.

## 6. Issues That Need to Be Addressed in GALM Deficiency

Several issues need to be addressed to elucidate the pathogenesis of GALM deficiency. First, the metabolic block has not directly been confirmed. The elevated levels of blood D-galactose, the impaired GALM enzymatic activities of mutant recombinant proteins and patient-derived proteins, and the instability of mutant GALM proteins have been shown as indirect evidence of GALM enzyme deficiencies [24]. The metabolic block in vivo, as determined by the elevated ratio of beta to alpha, has not been demonstrated. Considering that alpha/beta-D-galactose rapidly equilibrates in an aqueous solution (although the rate in cells is slower than that in aqueous solution [14,23]), it may be difficult to show the metabolic block of epimerization between the two galactose isoforms. In addition to confirming the metabolic block, the build-up of galactitol should be demonstrated as an accumulation of a toxic substrate in galactosemia, perhaps through urinalysis.

Second, other complications, apart from cataracts, remain unknown. In GALK1 deficiency, the neonatal incidence of transaminase elevation, bleeding diathesis, and encephalopathy are higher compared to those in the general population, as well as compared to the incidence of cataracts [16]. Since D-galactose, in a broader sense, accumulates in both GALK1 and GALM deficiency, complications observed in GALK1 deficiency may also be reported in some patients with GALM deficiency. Interestingly, one patient with GALM deficiency exhibited a transient elevation of transaminases and total bile acids during infancy, although not during the neonatal period.

Third, the natural history of individuals with biallelic pathological variants of *GALM* has also not been elucidated. Further research is needed to determine what proportion of individuals show no symptoms at all, including cataracts, without treatment; whether there is an association with symptoms other than cataracts; and whether this occurs outside of Japan.

## 7. Conclusions

A novel type of genetic galactosemia, GALM deficiency, was reported in the NBS for classic galactosemia in Japan. Due to the wide range of phenotypes, it is expected that a significant number of cases will remain undiagnosed, especially in countries and regions where NBS is unavailable or where galactose is not measured. Further studies are needed to clarify the natural history of GALM deficiency in humans, including the full spectrum of cataracts and other phenotypes.

## Figures and Tables

**Table 1:** Newborn screening for galactosemia in the US, Europe, and Japan.

	Japan	European Countries (51 Countries) [34]	US (53 States and Territories) ^1^	
Galactosemia screening	Nationwide	20 ^2^/51 (39.2%)	53/53 (100%)	
**Screening methods**			**First screen, first tier** ^3^	**First screen, second tier** ^3^
Total galactose	+	ND	12/53 (22.6%)	13/53 (24.5%)
Galactose	+	ND	0/53 (0%)	0/53 (0%)
Galactose-1-phosphate	+	ND	2/53 (3.8%)	0/53 (0%)
GALT activities	+	ND	37/53 (69.8%)	10/53 (18.9%)
Mutation analysis (*GALT*)	−	ND	0/53 (0%)	3/53 (5.7%)
No answer	NA	NA	6/53 (11.3%)	26/53 (49.1%)

^1^ https://www.newsteps.org/ (visited on 25 June 2021); ^2^ Three countries running pilot projects are included. ^3^ Multiple answers were possible. Abbreviations: ND = no data, and NA = not applicable.
